# Development and Psychometric Validation of a Multidimensional Clinical Coding Quality Assessment Instrument in Casemix Systems

**DOI:** 10.12688/f1000research.183114.3

**Published:** 2026-09-30

**Authors:** Kori Puspita Ningsih, Hartono Hartono, Nur Hafidha Hikmayani

**Affiliations:** 1 Doctoral Program in Public Health, Faculty of Medicine, Universitas Sebelas Maret, Surakarta, Central Java, 57126, Indonesia; 2 Department Of Medical Record and Health Information, Faculty of Health, Universitas Jenderal Achmad Yani Yogyakarta, Sleman, Daerah Istimewa Yogyakarta, 55294, Indonesia; 3 Department of Public Health, Faculty of Medicine, Universitas Sebelas Maret, Surakarta, Central Java, 57126, Indonesia; 4 Department of Pharmacology, Faculty of Medicine, Universitas Sebelas Maret, Surakarta, Central Java, 57126, Indonesia

**Keywords:** Clinical Coding Quality, Casemix, Coding Audit, Data Quality, Reimbursement Integrity

## Abstract

**Introduction:**

In casemix-based payment systems, clinical coding quality plays a critical role in determining data validity, reimbursement accuracy, and overall performance. Financial inefficiencies, skewed morbidity data, and claim denials might result from incorrect coding. However, the majority of studies pay little attention to thorough and context-specific evaluation, instead concentrating mostly on coding accuracy.

**Objective:**

The purpose of this study was to use the Indonesian Case-Based Groups (INA-CBG) as the empirical backdrop for the development and psychometric validation of a multidimensional instrument for evaluating clinical coding quality in casemix systems.

**Methods:**

An instrument development strategy based on ICD-10, ICD-9-CM, WHO coding guidelines, national casemix requirements, and results from coding audits was used to conduct a methodological investigation. The Content Validity Ratio (CVR) and Content Validity Index (I-CVI, S-CVI) were used to evaluate the content validity. Cohen’s Kappa was used to assess inter-rater reliability, while Cronbach’s alpha with 95% confidence intervals was used to quantify internal consistency. JASP was used for data analysis.

**Results:**

The Clinical Coding Quality Assessment Instrument for Casemix (CCQAI–CM) comprises seven conceptual domains operationalized through 11 assessment items: diagnosis documentation, procedure documentation, documentation support, coding compliance, coding accuracy, diagnosis reselection, and procedure reselection. The instrument demonstrated high content validity (S-CVI = 0.972), varying item-level inter-rater agreement, and very high overall internal consistency (Cronbach’s α = 0.982).

**Conclusion:**

The findings provide initial evidence of the content validity and reliability of the CCQAI–CM as a multidimensional framework for assessing clinical coding quality in casemix systems. Further validation across healthcare settings and against relevant casemix outcomes is warranted.

## Introduction

Casemix-based payment systems have emerged as a critical element of global healthcare funding reforms, seeking to enhance efficiency, transparency, and cost management while preserving service quality. Casemix systems depend significantly on standardised clinical data to accurately classify patients and provide equitable reimbursement by categorising them according to clinical parameters and resource utilisation. Central to this mechanism is clinical coding, which converts clinical diagnoses and procedures recorded in medical records into standardised categorisation systems, including the International Classification of Diseases (ICD) and procedure coding systems. ^1^

Clinical coding does not merely serve administrative or billing purposes; it forms the foundation for morbidity and mortality statistics, health service planning, quality measurement, and clinical research. ^2, 3^ Inaccurate or inconsistent coding compromises data validity, distorts epidemiological profiles, and may lead to inappropriate reimbursement decisions. Previous studies have demonstrated that coding errors can significantly affect case grouping, severity levels, and payment outcomes in casemix systems, thereby posing financial and governance risks for healthcare organizations. ^4– 6^

The quality of clinical coding is a multidimensional construct. While coding accuracy is often emphasized as the primary indicator, international health information management literature recognizes additional quality attributes, including completeness, reliability, timeliness, relevance, definition clarity, and legibility. ^7– 10^ These dimensions reflect the complex nature of coding activities, which require not only technical knowledge of classification systems but also the ability to interpret clinical documentation, apply coding rules consistently, and align coding decisions with regulatory requirements.

In practice, however, many coding quality assessments remain narrowly focused on accuracy alone, typically measured by comparing assigned codes against reference standards or expert consensus. ^11^ Such approaches may fail to capture critical issues related to rule application, documentation adequacy, and decision-making logic, particularly in casemix-based reimbursement environments. As a result, healthcare organizations may overlook systemic coding problems that contribute to claim rejections, pending payments, or inappropriate severity classification.

In Indonesia, the national health insurance system implements the Indonesian Case-Based Groups (INA-CBG) as the official casemix-based reimbursement mechanism. The INA-CBG system uses ICD-10 for diagnosis coding and ICD-9-CM for procedure coding. It also uses grouping algorithms and reimbursement rules set by the Ministry of Health and BPJS Kesehatan (BPJS Kesehatan, 2014; Regulation of the Minister of Health of the Republic of Indonesia Number 26 of 2021 concerning Guidelines for Indonesian Case Base Groups (INA-CBG), 2021). ^12, 13^ Within this system, clinical coders are required to apply not only international coding standards but also specific national regulations related to diagnosis selection, procedure coding, and reselection rules that directly influence casemix grouping outcomes.

Empirical evidence from coding audits in Indonesian hospitals reveals that coding quality issues extend beyond simple miscoding. Common problems include inaccurate application of general coding principles, failure to apply combination codes and dual coding rules appropriately, inconsistencies between codes and supporting clinical documentation, and incorrect reselection of primary and secondary diagnoses based on resource utilization. ^14– 16^ These issues frequently result in claim rejections or pending claims, indicating that coding quality directly affects reimbursement efficiency and financial sustainability.

The World Health Organization (WHO) states that the main diagnosis is the diagnosis that consumes the most resources during an episode of care and must be chosen in accordance with standard morbidity coding rules rules (Regulation of the Minister of Health of the Republic of Indonesia Number 26 of 2021 concerning Guidelines for Indonesian Case Base Groups (INA-CBG), 2021; WHO, 2010). Likewise, the national rule in Indonesia emphasises the necessity of proper diagnostic reselection and procedure coding for appropriate INA-CBG grouping and payment. ^12^ However, studies have demonstrated that coders often have difficulty following these criteria consistently, particularly when clinical documentation is limited or confusing. ^17– 19^

In addition, expert knowledge of ICD rules and judgement based on context is necessary for special circumstances such as the usage of combination codes, dual codes (dagger and asterisk), infectious agents, external causes of damage and treatment reselection. International research points out that these errors are among the most common and detrimental in casemix environments but are seldom rigorously examined in routine coding audits. ^5, 20, 21^

There are various international frameworks suggesting conceptual models for health data quality assessment, however there is little applicability to clinical coding in casemix systems. ^8, 10^ Existing instruments are typically not operationalised with the specific coding rules and reimbursement logic that limit their utility for actual audits and quality improvement efforts. In Indonesia, there is no standard comprehensive instrument that has been officially implemented to measure the quality of clinical coding in several aspects in accordance with the requirements of INA-CBG. ^8, 9^

Closing this gap is critical to improve health information governance and to achieve equitable reimbursement in casemix-based systems. A organised and thorough assessment instrument, combining international coding standards with national casemix laws, may give a better picture of the quality of coding and its ramifications. Such a tool can facilitate systematic audits, increase coder accountability, uncover core causes of coding errors, and direct focused initiatives to improve documentation and coding procedures.

Therefore, this work intends to construct a comprehensive tool for clinical coding quality evaluation in casemix systems, with the Indonesian INA-CBG implementation as empirical evidence. The study provides a new methodological contribution to the literature on clinical coding quality and casemix-based reimbursement systems, through operationalisation of multiple dimensions of coding quality, including general principles, combination and dual coding rules, documentation conformity, and diagnosis and procedure reselection.

## Methods

The study used a methodological research strategy for creating and psychometric testing of a multidimensional instrument for measuring clinical coding quality in casemix-based reimbursement systems. Methodological studies are often employed in health research to design, enhance and evaluate the validity and reliability of measurement tools. ^22^ In the present study, psychometric evaluation focused on content validity, inter-rater reliability, and internal consistency as initial measurement properties of the newly developed instrument.

The instrument was developed through a systematic, multi-phase process adhering to established protocols for instrument creation in health research, including item development, domain identification, and operationalization. ^23^ The instrument development process was methodical and comprised several stages:

a. Identifying Domains

• To identify the key dimensions of clinical coding quality relevant to casemix-based reimbursement, ICD-10, ICD-9-CM, WHO morbidity coding guidance, national INA-CBG requirements, and findings from previous clinical coding audits were reviewed. This process identified seven conceptual domains: (1) diagnosis documentation, (2) procedure documentation, (3) documentation support, (4) coding compliance, (5) coding accuracy, (6) diagnosis reselection, and (7) procedure reselection. The seven domains were operationalized into 11 assessment items (I1–I11): diagnosis documentation (I1–I2), procedure documentation (I3), documentation support (I4), coding compliance (I5–I7), coding accuracy (I8), diagnosis reselection (I9–I10), and procedure reselection (I11).
• The CCQAI–CM also includes a case-identification component used solely to record administrative information required for coding audit purposes. This component is not scored and is not considered one of the conceptual domains. Similarly, the final assessment represents a derived summary calculated from the applicable scored items rather than an additional domain or assessment item. Thus, the conceptual structure of the CCQAI–CM comprises an administrative component, seven conceptual domains operationalized through 11 items, and a derived overall assessment. Collectively, these domains conceptualize clinical coding quality as extending beyond code accuracy to include documentation adequacy, conformity with coding standards, and the appropriateness of diagnosis and procedure selection within the casemix context.

b. Item Generation and Operationalization

• The 11 assessment items constituted the initial item pool. No items were eliminated during the refinement process because each represented a conceptually and operationally distinct aspect of clinical coding quality. Refinement therefore focused on improving conceptual clarity, minimizing potential overlap, strengthening alignment with ICD coding standards and casemix requirements, and ensuring that each criterion could be objectively assessed using information available in the medical record. Each item was operationalized using predefined assessment criteria appropriate to its purpose, including yes/no, conforming/non-conforming, accurate/inaccurate, and appropriate/inappropriate. Items not relevant to a particular clinical case were classified as not applicable according to the predefined applicability rule. The resulting 11-item candidate instrument was subsequently subjected to expert content validation.

c. Incorporation of a Rule-Based Framework

The instrument employed rule-based logic derived from ICD coding standards, WHO morbidity coding guidance, and national casemix requirements to support standardized assessment across the 11 CCQAI–CM items.

Content validity was assessed using the Content Validity Ratio (CVR) and Content Validity Index (CVI) by a panel of 11 experts selected purposively according to predefined eligibility criteria. Eligibility criteria included: (1) a minimum master’s degree in a health-related field with an educational background in Medical Records and Health Information (RMIK), and (2) at least 10 years of professional practice and/or teaching experience in the field.

The panel comprised four experts from the Indonesian Professional Association of Medical Records and Health Information (PORMIKI), three RMIK academics, and four senior hospital practitioners representing casemix and medical-record departments in type A–D hospitals outside the Special Region of Yogyakarta. Their professional experience ranged from 14 to 33 years (mean 19.64 years).

Each expert independently evaluated the 11 items for relevance, clarity, and conformity with coding and casemix requirements. The Item-level Content Validity Index (I-CVI) was calculated as the proportion of favorable expert ratings, with ratings of 3 or 4 on a four-point scale considered favorable. The Scale-level Content Validity Index (S-CVI) was calculated as the mean I-CVI across items, with ≥0.90 considered indicative of excellent content validity. ^24, 25^ CVR was used to assess item essentiality. For a panel of 11 experts, the critical CVR was 0.636 (one-tailed α=0.05), corresponding to agreement by at least nine experts. The CVR (Avg) values reported in Table 1 represent the mean CVR across the assessed content dimensions of relevance, clarity, and conformity with coding and casemix requirements. Expert feedback was used to refine item wording and operational criteria, and all 11 items were retained.

**Table 1.** Content Validity of the Instrument (CVI).

No	Item	I-CVI (Avg)	CVR (Avg)	Interpretation
I1	Primary diagnosis documentation	1	1	Excellent
I2	Secondary diagnosis documentation	1	1	Excellent
I3	Procedure documentation	0,97	0,939	Excellent
I4	Supporting clinical information	0,97	0,939	Excellent
I5	Specialist conformity	0,909	0,818	Acceptable
I6	ICD-10 coding	1	1	Excellent
I7	ICD-9-CM coding	1	1	Excellent
I8	Diagnosis accuracy	1	1	Excellent
I9	Primary reselection	0,909	0,879	Acceptable
I10	Secondary reselection	0,909	0,879	Acceptable
I11	Procedure reselection	0,909	0,879	Acceptable
S-CVI = 0.972 (Excellent)				

*Note:* CVR (Avg) represents the mean CVR across the assessed content dimensions. For 11 experts, the critical CVR value was 0.636 (one-tailed α = 0.05).

Two different ways were used to check reliability:

a. Reliability between raters

Cohen’s Kappa was used to assess inter-rater reliability because the CCQAI–CM items produce categorical ratings independently assigned by two raters. Kappa measures agreement beyond that expected by chance and was therefore considered appropriate for assessing item-level agreement. The classification criteria proposed by Landis and Koch ^26^ were used for interpretation: values below 0.40 indicated poor to fair agreement, 0.41–0.60 moderate agreement, 0.61–0.80 substantial agreement, and values above 0.80 almost perfect agreement.

b. Consistency throughout the organization

Cronbach’s alpha was used to assess the internal consistency of the multi-item instrument. ^27^ Values of ≥0.70 were considered acceptable, ≥0.80 indicated good reliability, and ≥0.90 indicated excellent internal consistency. Because the seven CCQAI–CM domains contain only one to three items, including several single-item domains, domain-specific alpha coefficients were not considered appropriate for interpretation.

The instrument was utilized on a sample of inpatient medical records designated for coding audit purposes. Two trained raters used the devised tool to look at each record separately. Discharge summaries, clinical notes, operation reports, laboratory findings, and radiology reports were all used as data sources.

We used JASP statistical software to analyze the data. The analyses comprised content validity assessment using CVR, I-CVI, and S-CVI; inter-rater reliability assessment using Cohen’s Kappa for each item; and internal consistency assessment using Cronbach’s alpha. Reliability estimates were reported with 95% confidence intervals. Together, these analyses provided initial evidence regarding the content validity and reliability of the CCQAI–CM.

## Results

### Content validity

The content validity analysis indicated strong expert agreement regarding the relevance and essentiality of the instrument items. The I-CVI values presented in Table 1 ranged from 0.909 to 1.000, while the CVR (Avg) values ranged from 0.818 to 1.000. All items therefore remained above the applicable content-validity criteria and were retained. Items related to specialist conformity and reselection (I5, I9–I11) showed relatively lower values than the other items, suggesting greater variation in expert judgment for components requiring contextual clinical and casemix interpretation.

The Scale-level Content Validity Index (S-CVI) was 0.972, exceeding the recommended threshold of 0.90 and supporting strong overall content validity of the 11-item instrument.

### Inter-rater reliability

Inter-rater reliability analysis demonstrated varying levels of agreement across items, as presented in Table 2. Cohen’s Kappa values ranged from 0.403 to 0.802, indicating agreement levels from moderate to almost perfect.

**Table 2.** Inter-rater reliability of the instrument using Cohen’s Kappa.

Item	Kappa (κ)	SE	95% CI Lower	95% CI Upper	Interpretation
I1	0,802	0,106	0,593	1,000	Almost Perfect
I2	0,537	0,15	0,243	0,832	Moderate
I3	0,714	0,131	0,457	0,972	Substantial
I4	0,429	0,169	0,098	0,759	Moderate
I5	0,67	0,132	0,41	0,929	Substantial
I6	0,507	0,161	0,191	0,823	Moderate
I7	0,464	0,162	0,147	0,782	Moderate
I8	0,667	0,136	0,401	0,933	Substantial
I9	0,795	0,112	0,575	1,000	Substantial
I10	0,426	0,171	0,091	0,761	Moderate
I11	0,403	0,174	0,061	0,744	Moderate

The greatest degree of concordance was noted for primary diagnostic documentation (I1; κ = 0.802), indicating robust consistency among raters in evaluating distinctly defined and objective documentation components. Likewise, elements pertaining to coding precision and adherence (I3, I5, and I8) exhibited significant concordance, indicating that rule-based coding components often yield more uniform assessments.

Conversely, less agreement was noted in questions requiring more intricate clinical judgment, including procedure reselection (I11; κ = 0.403) and diagnostic reselection (I9–I10). The findings suggest that diversity among raters is more significant in domains necessitating interpretative decision-making than in those involving simple rule application.

The average Kappa value was 0.583, signifying high agreement among raters ( Table 3). This indicates that although the instrument offers a systematic evaluation framework, certain aspects of clinical coding quality intrinsically require subjective clinical judgment.

**Table 3.** Summary of reliability analysis.

Measure	Value	SE	95% CI Lower	95% CI Upper	Interpretation
Mean Cohen’s Kappa	0,583	–	–	–	Moderate–Substantial
Cronbach’s Alpha	0,982	0,008	0,966	0,997	Excellent

### Internal consistency

The internal consistency analysis revealed an exceptional degree of reliability for the CCQAI–CM instrument. Table 3 demonstrates that Cronbach’s alpha coefficient was 0.982 (95% CI: 0.966–0.997), signifying an exceptionally high level of internal consistency among the items. All items demonstrated robust item–rest correlations, indicating that each item significantly contributes to the overall construct. Nonetheless, some items had significantly high inter-item correlations, suggesting possible redundancy among conceptually analogous markers. Collectively, the inter-rater reliability data indicate that the instrument exhibits robust internal consistency, although discrepancies in inter-rater agreement may signify changes in clinical judgment rather than inherent deficiencies of the instrument.

### Structure of the CCQAI–CM instrument

Table 4 delineates the structural and operational framework of the CCQAI–CM. The instrument comprises an administrative case-identification component, seven conceptual domains operationalized through 11 assessment items, and a derived overall assessment. The case-identification component is not scored, and the final assessment is not an additional domain or item; rather, it summarizes the applicable scored items. For terminology, coding accuracy refers to correctness of assigned codes relative to the documented condition; coding compliance refers to adherence to applicable coding standards and rules; clinical consistency refers to whether the diagnosis is supported by available clinical evidence; and specialist conformity refers to consistency between the diagnosis and documented specialist findings.

**Table 4.** CCQAI–CM (Clinical coding quality assessment instrument for casemix): A multidimensional assessment form for evaluating clinical coding quality, documentation conformity, and casemix-based decision logic.

Section	Domain	Item	Assessment focus	Criteria	Applicability	Rating
A	Case Identification	–	Patient & case identification	Completeness of case information	Applicable	Complete/Incomplete
B	Diagnosis Documentation	I1	Primary diagnosis documentation	Documented in discharge summary	Applicable	Yes/No
		I2	Secondary diagnosis documentation	All relevant diagnoses documented	Applicable	Yes/No
C	Procedure Documentation	I3	Procedure documentation	Procedures recorded in medical record	Conditional	Yes/No
D	Documentation Support	I4	Clinical consistency	Diagnosis supported by clinical evidence	Applicable	Conforming/Non-conforming
E	Coding Compliance	I5	Specialist conformity	Diagnosis consistent with specialist findings	Applicable	Conforming/Non-conforming
		I6	ICD-10 coding	Diagnosis coded using correct ICD-10	Applicable	Accurate/Inaccurate
		I7	ICD-9-CM coding	Procedure coded using correct ICD-9-CM	Conditional	Accurate/Inaccurate
F	Coding Accuracy	I8	Code accuracy	Codes reflect documented condition	Applicable	Accurate/Inaccurate
G	Diagnosis Reselection	I9	Primary diagnosis reselection	Based on resource utilization	Applicable	Appropriate/Inappropriate
		I10	Secondary diagnosis reselection	Reflects severity/comorbidity	Applicable	Appropriate/Inappropriate
H	Procedure Reselection	I11	Procedure reselection	Reflects performed interventions	Conditional	Appropriate/Inappropriate
I	Final Assessment	–	Overall coding quality	Integrated evaluation across domains	Applicable	Good/Fair/Poor

*Note:* Sections A and I are organizational components for case identification and the derived final assessment, respectively; the seven conceptual domains are presented in Sections B–H and are operationalized through items I1–I11.

### Scoring and interpretation of the CCQAI–CM

The structure and operational framework of the CCQAI–CM, including its domains, items, applicability status, and rating categories, are presented in Table 4. Items I3, I7, and I11 are conditional items and should only be assessed when procedures or medical interventions are relevant to the case. Positive ratings are scored as 1, whereas negative ratings are scored as 0. All applicable items are equally weighted in the overall score to maintain a transparent and readily interpretable audit measure; differential weighting was not applied in this initial development study because empirical evidence for domain-specific weights has not yet been established. A conditional item is classified as not applicable (N/A) when the relevant procedure or intervention is absent from the case and therefore cannot be meaningfully assessed. For example, I3 and I11 are N/A when no procedure or intervention is documented, while I7 is N/A when no procedure requiring ICD-9-CM coding is relevant to the case. N/A items are excluded from the denominator rather than scored as negative responses. For example, if eight of the 11 items are applicable and seven receive a positive rating, the final score is 7/8 × 100% = 87.5%.

$$\text{Final Score}=\frac{\text{Total Positive Score}}{\text{Number of Applicable Items Assessed}}\;x\;100\%$$

The final score is then interpreted into three categories, as shown in Table 5.

**Table 5.** Interpretation categories of the CCQAI–CM final score.

Score	Category
≥85%	Good
70–84%	Fair
<70%	Poor

The final score is interpreted using three pragmatic categories: ≥85% (Good), 70–84% (Fair), and <70% (Poor). In this initial instrument-development study, these thresholds function as operational interpretive bands rather than empirically optimized diagnostic cut-offs. Accordingly, their classification performance should be evaluated in future studies using external clinical and casemix outcomes. A score of ≥85% indicates that most applicable criteria are fulfilled; 70–84% indicates that selected areas require improvement; and <70% indicates broader deficiencies that may warrant review of documentation, coding practice, and related quality-improvement measures.

### Summary of psychometric properties

The combined results of content validity, inter-rater reliability, and internal consistency indicate that the CCQAI–CM demonstrates strong psychometric performance. The high S-CVI confirms the relevance and representativeness of the instrument items, while of the item-level Kappa values indicate varying levels of agreement between raters. In addition, the high Cronbach’s alpha reflects strong internal coherence among items. Together, these findings suggest that the instrument is both conceptually valid and practically reliable for assessing clinical coding quality. Further evaluation of additional measurement properties and application across broader clinical settings would strengthen the evidence for its continued use and development.

## Discussion

### 1. Content validity and conceptual strength

The findings demonstrate that the CCQAI–CM instrument possesses exceptional content validity, as indicated by a high S-CVI score (0.972), reflecting substantial consensus among experts concerning the relevance and representativeness of the questions. The concurrent application of I-CVI and CVR enhances the validation process by assessing the relevance and necessity of each item, ensuring that each item significantly contributes to the concept of clinical coding quality. ^28, 29^

Specifically, items related to speciality conformance and reselection suitability had somewhat lower CVR values. These differences are likely to be due to differences in opinion among expert coders on complicated clinical coding judgements that need clinical judgement rather than straightforward rule-based assessment. This corresponds to ^29^ original premise that disagreement among experts is more common in fields of interpretative complexity than in those with standardised standards.

Importantly, this finding aligns with real-world coding audit practices, where disagreement among auditors often arises not from ambiguity in coding rules, but from the interpretative complexity of clinical documentation and decision-making processes. In practice, auditors need to pull together information from many sections of the medical record (e.g., discharge summaries, progress notes, test data, procedure reports) to identify the most appropriate diagnosis and assignment of codes. Variability might arise from inconsistent or inadequate documentation, or documentation that lacks sufficient clinical clarity, forcing auditors to exercise professional judgement in understanding the clinical context. Variations in training, experience, and familiarity with coding guidelines may also impact the way coders interpret and prioritise clinical data, as described within clinical documentation improvement (CDI) frameworks, resulting in discrepancies in coding outcomes despite codified rules. ^30^ This emphasises that assessing the accuracy of coding is not simply a technical quality assurance exercise, but a cognitive exercise requiring clinical judgement and contextual decision making.

### 2. Inter-rater reliability

Inter-rater reliability analysis demonstrated moderate to substantial concordance among items, with increased consistency noted in documentation and rule-based coding elements. The findings demonstrate that organised and standardised coding features are perceived more consistently among raters, highlighting their objective nature. ^31, 32^

In contrast, diminished consensus on reselection-related items shows the intrinsic complexity of clinical coding judgements that necessitate contextual interpretation, including severity evaluation and resource allocation. Comparable results have been documented in research investigating discrepancies in coding techniques, with greater disagreement evident in instances necessitating interpretative clinical reasoning. ^5, 6^ This pattern reflects findings in coding audit literature, where variability is particularly evident in areas requiring contextual interpretation, such as secondary diagnosis capture and rule-based reselection decisions. ^18, 33^ The observed Kappa values align with known benchmarks for clinical audit investigations, ^26^ signifying that the instrument exhibits sufficient reliability for practical application.

### 3. Internal consistency

Excellent internal consistency is shown by the Cronbach’s alpha value of 0.982, which is indicative of good coherence among the items of the CCQAI–CM. ^34^ Very high alpha values can sometimes be a sign of item redundancy, but in the case of this instrument, the high inter-item correlations may reflect conceptual and practical interdependence among clinical coding quality characteristics, while also warranting consideration of possible item similarity. Documentation quality is a core aspect that directly impacts coding compliance, coding accuracy and diagnosis/procedure reselection. This interpretation is reinforced by clinical documentation improvement literature, which emphasises that accurate and thorough clinical recording is critical to correct coding, compliance, payment integrity, audit preparedness, and reliable health data reporting. ^30, 35^

Incomplete or imprecise documentation might influence the implementation of ICD regulations, the choice of primary and secondary diagnoses and the validity of the coded data in casemix-based reimbursement systems. Previous research has established a close relationship between the quality of coding and the validity of administrative data and the completeness and accuracy of clinical documentation, and the aim of clinical documentation improvement initiatives is to enhance the specificity of documentation, the accuracy of coding, and data quality. ^5, 30, 36^ Thus, the high internal consistency found in this study suggests the integrated nature of the clinical coding process and not simply repetition of items.

### 4. Integration of psychometric findings

The combination of excellent internal consistency and moderate inter-rater reliability highlights the multidimensional and interpretative nature of clinical coding quality assessment. While Cronbach’s alpha reflects strong internal coherence among the CCQAI–CM items, Kappa values capture variability in rater interpretation. This distinction is well established in psychometric theory, where different reliability measures assess different aspects of measurement quality. ^37^ Therefore, the observed difference between internal consistency and inter-rater agreement should not be viewed as contradictory, but rather as complementary evidence regarding the performance of the instrument.

The very high internal consistency observed in this study may reflect the conceptual and operational interdependence among clinical coding quality dimensions, although possible item similarity should also be considered. In clinical coding practice, documentation completeness, clinical support, coding compliance, coding accuracy, and diagnosis/procedure reselection are not isolated processes. However, they are part of a workflow, where the quality of one element immediately impacts the next. Incomplete or inconsistent clinical documentation may hinder the coder’s capacity to appropriately apply ICD guidelines, identify the correct primary and secondary diagnoses, and confirm that coded data accurately reflect the patient’s clinical status. This interpretation is consistent with clinical documentation improvement literature stressing the necessity of precise, thorough and specific documentation for coding accuracy, compliance, reimbursement integrity, audit readiness and accurate health data reporting. ^5, 30^

Kappa values were moderate, indicating that reviewers need to consistently assess and interpret multiple items clinically. Discrepancies in agreement are expected, particularly for items involving clinical consistency, diagnosis reselection and relevance of secondary diagnosis and procedure reselection since these require the coders to assess clinical evidence, follow coding guidelines and prioritise diagnostic or procedural importance in the casemix context. The variation in kappa values observed is likely to reflect variation in clinician judgement and coder interpretation rather than limitations of the instrument itself. This interpretation is consistent with health information and data quality frameworks that suggest data accuracy is determined by more than structural consistency, standardised formats, coding systems or set regulations. Their reliability is a function of the consistency with which the data definitions are interpreted and applied in practice by users. Indeed, even with standardised data items, variability may occur due to differences in clinical documentation quality, coder training, experience and knowledge of coding standards. This is particularly true for casemix-based reimbursement systems, as coding decisions affect the quality of the administrative data, outcome grouping and financial implications. ^35, 36, 38^

The psychometric results indicate that the CCQAI–CM exhibits robust internal consistency and effectively addresses aspects of clinical coding that necessitate interpretative judgement. The elevated Cronbach’s alpha indicates strong conceptual integration of the instrument, while the Kappa results underscore the necessity for coder calibration, systematic training, and enhancements in clinical documentation to promote consistency in instrument use among raters and contexts. Accordingly, the current findings are best interpreted as initial evidence supporting the content validity and reliability of the CCQAI–CM rather than definitive evidence that the instrument has completed the full validation pathway.

### 5. Domain-Based interpretation of findings

A domain level analysis gives more insight into the performance of the instrument. Unsurprisingly, documentation and coding compliance domains had higher levels of agreement, as they rely on structured and standardised criteria. These areas correspond with the processes involved in coding audits which verify completeness of documentation and adherence to coding standards. ^11, 30^ This is consistent with contemporary coding audit frameworks, which emphasize that coding quality extends beyond simple code matching to include documentation validity and rule application. ^8, 33^

In contrast, reselection domains showed lower agreement, pointing to their reliance on clinical judgement and context-specific interpretation. In such domains, the evaluator needs to combine multiple factors, such as severity of diagnosis, use of resources, and clinical relevance, which naturally leads to variability. ^39, 40^ Critically this confirms that clinical coding quality is not simply technical but a hybrid construct that combines procedural accuracy and clinical reasoning. Finally, the addition of an applicability component adds flexibility when some items are not relevant, thus enhancing the usability of the instrument across different clinical settings.

### 6. Novelty and contribution

The CCQAI–CM represents a potentially useful contribution by providing a multidimensional, rule-based and context-sensitive framework for assesing clinical coding quality in casemix systems. This instrument, unlike traditional methods that focus on coding correctness or inter-coder agreement, contains documentation completeness, coding compliance, coding accuracy and reselection logic based on casemix principles. Previous research predominantly considers accuracy measurements or agreement statistics as critical indicators of the quality of codes, often overlooking upstream issues such as the sufficiency of documentation and adherence to coding standards which have a significant impact on the coding results. ^5, 31, 32^

From a data quality perspective, the instrument is consistent with and builds upon multidimensional data quality frameworks that emphasise “fitness for use” as a central concept. ^36, 38^ Here, data must not only be syntactically correct but also semantically consistent, clinically valid, and consistent with decision rules that govern classification and payment. Moreover, the inclusion of documentation conformity as a core assessment domain further strengthens the link between clinical documentation and coding quality, bolstering the principle that the validity of administrative data is based on the consistency between coded data and verifiable clinical evidence. ^35, 41, 42^

From a governance perspective, the CCQAI–CM assists in conceptualising coding quality as a health information governance mechanism, rather than a mere technical outcome. Information governance systems focus on accountability, standardisation, transparency and auditability at all stages of the health information lifecycle. ^7, 35, 43^ The development of the instrument according to rules and standards is in accordance with these principles, allowing for systematic monitoring of the instrument, traceability of coding decisions and systematic implementation of coding standards and national casemix rules. The instrument also contributes to reimbursement integrity theory by directly linking coding quality assessment with casemix grouping logic and principles of resource utilisation. Casemix systems are built on the assumption that diagnostic and procedural codes are proxies for patient complexity and intensity of care. ^40^ The CCQAI–CM operationalises reimbursement integrity at the micro-level of coding decisions. It conceptualises the link between coding practices and payment system sustainability through assessment of the appropriateness of diagnosis and procedure reselection.

In practical terms, the instrument provides healthcare organisations with a structured way to systematically evaluate coding performance and identify underlying weaknesses in documentation and coding workflows. It reveals patterns of non-compliance, documentation gaps and misapplication of rules and facilitates targeted interventions such as coder retraining, clinician documentation improvement and audit-driven corrective actions. These applications align with modern coding audit approaches that aim to improve revenue integrity and compliance readiness. ^44^

These contributions together position the CCQAI-CM not just as an audit tool but as a integrated framework linking rule-based coding systems to clinical reasoning. This integrated framework provides a more realistic and operationalisable approach to measuring the quality of clinical coding in casemix-based healthcare systems and also establishes the foundation for future research into coding governance, audit effectiveness and quality of administrative data.

### Scope, limitations, and future validation

This study focused on instrument development and initial psychometric evaluation and did not examine associations between CCQAI–CM scores and external casemix outcomes such as pending claims, claim rejection, grouping changes, reimbursement discrepancies, or financial impact. These outcomes are important targets for subsequent criterion-related and predictive validation. Broader validation across multiple hospitals, clinical specialties, and healthcare settings is also required before generalized implementation can be recommended. The equal weighting of items and the current score-interpretation thresholds should likewise be examined empirically in future validation studies.

## Conclusion

This study developed a comprehensive and multidimensional instrument for assessing clinical coding quality within casemix-based reimbursement systems, using the Indonesian INA-CBG implementation as the empirical context. By integrating international coding standards, WHO coding rules, and national casemix regulations, the CCQAI–CM addresses the limitations of conventional accuracy-based assessments by incorporating documentation completeness, coding compliance, coding accuracy, and diagnosis/procedure reselection logic within a unified framework.

The results indicate that the quality of clinical coding is related not only to the correct assignment of codes, but also to the adequacy of the clinical documentation, the consistency in the application of coding standards and the appropriateness of the reselection of diagnoses and procedures. The dimensions reflect both rule-based processes and clinical reasoning, showing the complexity of coding practices in casemix systems. Thus, the CCQAI–CM provides initial evidence of content validity and reliability as a multidimensional framework linking coding practices to data quality, health information governance, and reimbursement integrity. Further validation across healthcare settings and against relevant casemix outcomes is warranted to extend evidence for the instrument’s measurement and practical performance.

## Ethical considerations

This study was reviewed and approved by the Research Ethics Committee of Universitas Jenderal Achmad Yani Yogyakarta, Indonesia (Approval No: Skep/001/Kep/I/2024). Approval was obtained before data collection. The study used anonymised health information and instrument validation data, and all procedures were conducted in accordance with ethical principles for health information research. The approval was obtained from Universitas Jenderal Achmad Yani Yogyakarta because the research was initiated and administratively managed through that institution, while the doctoral affiliation of the first author is Universitas Sebelas Maret.

## Informed consent

Written informed consent was obtained from all expert panel members and raters who participated in the content validity and reliability assessment. Participants were informed about the purpose of the study, their role in the validation process, the voluntary nature of participation, and the confidentiality of their responses. The clinical record data used for coding quality assessment were anonymised before analysis, and no directly identifiable patient information was included in the dataset or manuscript. Individual patient consent was not required because the study used anonymised secondary health record data and did not involve direct patient contact, as approved by the ethics committee.

## Data Availability

*Zenodo:* Baseline dataset for the development and psychometric evaluation of the Clinical Coding Quality Assessment Instrument for Casemix (CCQAI–CM). https://doi.org/10.5281/zenodo.20223103. ^45^ This repository contains the baseline dataset used in the development, content validity assessment, inter-rater reliability analysis, and internal consistency testing of the CCQAI–CM instrument. Data are available under the terms of the Creative Commons Attribution 4.0 International (CC BY 4.0) license.
