# Supplementary material for: Development and Psychometric Validation of a Multidimensional Clinical Coding Quality Assessment Instrument in Casemix Systems
Source: F1000Res. 2026 Sep 30;15:1138. Originally published 2026 Jul 11. [Version 3] doi: 10.12688/f1000research.183114.3 (PMC13594603; doi:10.12688/f1000research.183114.3)
Supplement: Peer review history

# Reviewer report: Reviewer response for version 2

**DOI:** 10.5256/f1000research.209477.r520064

**Published:** 2026-09-21

**Authors:** Yafi Sabila Rosyad

**Affiliations:** 1 Mental health nursing , Universitas Bhakti Husada Indonesia, Kuningan, Malaysia

I consider the manuscript substantially improved and suitable for further consideration after **minor revision**. However, several points would benefit from additional clarification to improve methodological transparency and the interpretability of the reported psychometric findings.

1. **Characteristics of the medical-record sample**
  Please provide more detailed information regarding the medical-record sample used for the inter-rater reliability and internal consistency analyses. In particular, please report the total number of medical records, sampling method, study period, hospital characteristics, and relevant clinical or case-mix characteristics of the records included. It would also be helpful to report the number or proportion of records for which each conditional item (I3, I7, and I11) was applicable. Please clarify whether the same set of medical records was used for all reliability analyses.
2. **Handling of non-applicable items in the reliability analysis**
  Because I3, I7, and I11 are conditional items, please clarify how non-applicable (N/A) responses were handled when calculating Cronbach’s alpha. Please indicate the number of observations included in the analysis and whether complete-case, pairwise, or another approach was used. If available, reporting item-rest correlations and/or alpha-if-item-deleted values would further support the interpretation of the very high Cronbach’s alpha (α = 0.982). This is particularly relevant because a very high alpha may also reflect substantial similarity among items.
3. **Conceptual distinction between I6 and I8**
  Please clarify the conceptual and operational distinction between I6 (“Diagnosis coded using correct ICD-10”) and I8 (“Codes reflect documented condition”). At present, these two items appear potentially overlapping. Providing explicit operational definitions and, preferably, an example of a coding situation in which I6 would receive a different rating from I8 would strengthen the instrument's conceptual clarity.
4. **Conceptual distinction between I4 and I5**
  Similarly, please further clarify the distinction between I4 (clinical consistency) and I5 (specialist conformity). Please specify the type of evidence that should be considered when scoring each item and provide an example of a situation in which these two items could result in different ratings.
5. **Clarification of CVR (Avg)**
  Please clarify how the reported “CVR (Avg)” values in Table 1 were calculated. Specifically, please identify the content dimensions assessed for CVR and, if possible, provide the underlying CVR values for each dimension before calculating the average. This would make the content validation procedure more transparent and reproducible.
6. **Applicability of conditional items and final scoring**
  Please provide concrete examples showing when I3, I7, and I11 should be classified as non-applicable. In addition, please explain more explicitly how an N/A item affects the denominator and the resulting final percentage score. A short example calculation may be useful for readers who intend to apply the instrument in practice.
7. **Interpretation of the mean Cohen’s kappa**
  Please reconsider the wording used to interpret the mean Cohen’s kappa (κ = 0.583). According to the classification presented in the Methods section, a value between 0.41 and 0.60 falls within the “moderate” range. Therefore, the expression “moderate–substantial” appears slightly inconsistent with the classification provided. Please standardize the terminology throughout the manuscript.
8. **Consistency in describing the level of validation**
  The Discussion appropriately acknowledges that the current study provides initial evidence of content validity and reliability and that further validation is required. However, the Conclusion appears to use stronger wording by describing the CCQAI–CM as a “psychometrically validated measurement tool.” Given that construct validity, factorial validity, criterion validity, convergent/discriminant validity, and test–retest reliability were not assessed in the present study, I recommend aligning the Conclusion more closely with the cautious interpretation in the Discussion. The instrument may more appropriately be described as having **initial evidence of content validity and reliability**, pending further validation.
9. **Moderation of novelty-related statements**
  Finally, some statements describing the CCQAI–CM as a “major contribution” and a “complete conceptual model” could be moderated slightly. The instrument is potentially valuable, but the current study represents an initial validation stage. More measured wording would better reflect the evidence currently available while still highlighting the potential contribution of the instrument.

Is the work clearly and accurately presented and does it cite the current literature?

Yes

If applicable, is the statistical analysis and its interpretation appropriate?

Yes

Are all the source data underlying the results available to ensure full reproducibility?

Yes

Is the study design appropriate and is the work technically sound?

Yes

Are the conclusions drawn adequately supported by the results?

Yes

Are sufficient details of methods and analysis provided to allow replication by others?

Yes

Reviewer Expertise:

Health science

I confirm that I have read this submission and believe that I have an appropriate level of expertise to confirm that it is of an acceptable scientific standard.

# Response

**Published:** 2026-09-22

**Authors:** Kori Puspita Ningsih

**Affiliations:** Public Health, Doctoral Program in Public Health, Faculty of Medicine, Universitas Sebelas Maret, Surakarta, 57126, Indonesia

Thank you very much for your careful and constructive review of our manuscript. We sincerely appreciate your valuable suggestions, which have helped us further improve the clarity, methodological transparency, and consistency of the manuscript. We have carefully considered all of your comments and have made targeted revisions and clarifications where appropriate, particularly regarding the applicability and scoring procedures, conceptual distinctions among items, content validity, inter-rater reliability, and the level of validation and novelty claims. Where additional information or analyses were not available from the original study data or were beyond the scope of the present study, we have clarified the existing procedures and appropriately moderated our interpretation. A detailed point-by-point response will accompany the revised manuscript. We sincerely appreciate your thoughtful recommendations and believe that these revisions have further strengthened the manuscript while preserving the original scope and design of the study.

# Reviewer report: Reviewer response for version 2

**DOI:** 10.5256/f1000research.209477.r521375

**Published:** 2026-09-17

**Authors:** Yastori Yastori

**Affiliations:** 1 APIKES IRIS, Padang, Indonesia

I have reviewed the revised manuscript and the authors’ responses to my previous comments. I am satisfied that the revisions have adequately addressed the issues and suggestions raised in my review. The manuscript has been improved accordingly, and I have no further comments or recommendations for revision. I therefore consider the manuscript suitable to proceed to the next stage of the indexing process.

Is the work clearly and accurately presented and does it cite the current literature?

Partly

If applicable, is the statistical analysis and its interpretation appropriate?

Partly

Are all the source data underlying the results available to ensure full reproducibility?

Partly

Is the study design appropriate and is the work technically sound?

Partly

Are the conclusions drawn adequately supported by the results?

Yes

Are sufficient details of methods and analysis provided to allow replication by others?

Partly

Reviewer Expertise:

Pending Claim, Coding ICD-10, ICD-9-CM, Design Web for early warning detection of pending claim, etc.

I confirm that I have read this submission and believe that I have an appropriate level of expertise to confirm that it is of an acceptable scientific standard.

# Response

**Published:** 2026-09-19

**Authors:** Kori Puspita Ningsih

**Affiliations:** Public Health, Doctoral Program in Public Health, Faculty of Medicine, Universitas Sebelas Maret, Surakarta, 57126, Indonesia

Thank you very much for your careful review and constructive feedback. We sincerely appreciate your positive assessment and are pleased that the revisions have satisfactorily addressed your previous comments. Your valuable suggestions have helped us improve the clarity and quality of the manuscript. We are truly grateful for your time, expertise, and kind consideration.

# Reviewer report: Reviewer response for version 1

**DOI:** 10.5256/f1000research.202121.r510484

**Published:** 2026-08-25

**Authors:** Yastori Yastori

**Affiliations:** 1 APIKES IRIS, Padang, Indonesia

**Recommendation: MAJOR REVISION**

 The manuscript states that seven principal domains were identified, yet Table 4 presents multiple sections/domains (A–H) and 11 assessment items, in addition to an overall final assessment. The authors should clearly define the conceptual hierarchy of the CCQAI–CM, including the distinction between domains, sections, items, and the final assessment.

 The manuscript should provide a detailed description of the item-generation process, including the initial item pool, criteria for item inclusion/exclusion, sources of each item, expert involvement during item development, and whether qualitative methods were used to establish contextual relevance.

 The authors should report the number of experts, professional backgrounds, years of experience, expertise criteria, recruitment method, and geographical/institutional diversity of the expert panel. The manuscript should also provide the rationale or reference for the CVR acceptance threshold based on the actual number of experts.

 The psychometric evaluation is primarily based on content validity, inter-rater reliability, and internal consistency. No evidence is presented for construct validity, criterion-related validity, convergent validity, discriminant validity, test-retest reliability, or factorial structure. The authors should either expand the validation analysis or moderate the claim of “psychometric validation” throughout the manuscript.

 The reported alpha of 0.982 is exceptionally high and may indicate substantial item redundancy. Furthermore, the CCQAI–CM appears conceptually multidimensional. The authors should provide domain-specific reliability estimates and discuss whether a single overall alpha is conceptually appropriate.

 The cut-off points of ≥85%, 70–84%, and <70% require stronger empirical or theoretical justification. The authors should explain how these thresholds were derived and, ideally, provide sensitivity analyses or empirical evidence supporting their classification accuracy.

1. Clarify the conceptual structure of the CCQAI–CM because the manuscript describes seven domains, whereas Table 4 presents multiple sections and 11 items.
2. Provide a transparent description of the item-generation process, including the initial item pool, item reduction process, theoretical framework, and expert involvement.
3. Provide detailed characteristics and sample size of the expert panel used for content validation and justify the CVR threshold based on the number of experts.
4. The claim of “psychometric validation” should be reconsidered because construct validity, criterion validity, factorial validity, test-retest reliability, and other forms of validity are not demonstrated.
5. The extremely high Cronbach's alpha (0.982) requires deeper analysis, including domain-specific reliability and assessment of possible item redundancy.
6. Provide complete information regarding the medical-record sample, sampling strategy, sample-size calculation, clinical characteristics, and distribution of applicable/non-applicable items.
7. Provide empirical or theoretical justification for the ≥85%, 70–84%, and <70% scoring thresholds.
8. Discuss the implications of equal weighting of all items and whether different domains should contribute equally to the overall coding-quality score.
9. The authors should demonstrate whether CCQAI–CM scores are associated with meaningful casemix outcomes, such as claim rejection, pending claims, grouping changes, reimbursement discrepancies, or financial impact.
10. Further validation across multiple hospitals and different clinical settings is required before broad implementation can be recommended.
11. Terminology should be standardized throughout the manuscript.   
12. The distinction between “coding accuracy”, “coding conformity”, and “coding compliance” should be explicitly defined.  
13. The operational definition of “clinical consistency” needs greater clarity.  
14. The operational definition of “specialist conformity” should be explained.  
15. The manuscript should clarify how disagreements between raters were resolved.
16. Training/calibration procedures for the two raters should be reported in detail.
17. Confidence intervals should be reported consistently for all reliability estimates.  
18. The rationale for selecting Cohen's Kappa should be explained.  
19. The applicability rule should be described with examples.  
20. The manuscript should distinguish between instrument development and instrument validation.

The article addresses an important research problem and presents a relevant framework, a practical instrument, strong content validity, and an effort to integrate coding rules, clinical documentation, and casemix decision logic, but the evidence supporting the instrument’s psychometric validity is not yet comprehensive. The domain structure needs to be clarified, the characteristics of the sample and expert panel need to be reported in greater detail, the exceptionally high Cronbach’s alpha requires further investigation for potential item redundancy, and the scoring thresholds lack sufficient empirical justification. Most importantly, ***the study does not yet demonstrate the relationship between the instrument’s scores and actual casemix outcomes, such as pending claims, claim rejection, changes in case grouping, or financial impact*** *.*

 Therefore, the article has good indexing potential, but I would not yet consider the CCQAI–CM a definitively validated instrument. It would be more appropriate to describe it as an instrument that has undergone initial content-validity and reliability evaluation.

 From a researcher’s perspective, this final research gap represents the most interesting opportunity for further investigation: transforming the CCQAI–CM from merely an instrument for measuring coding quality into ***a predictive tool for pending-claim risk based on multidimensional coding quality and Explainable Artificial Intelligence (XAI)*** *.*

Is the work clearly and accurately presented and does it cite the current literature?

Partly

If applicable, is the statistical analysis and its interpretation appropriate?

Partly

Are all the source data underlying the results available to ensure full reproducibility?

Partly

Is the study design appropriate and is the work technically sound?

Partly

Are the conclusions drawn adequately supported by the results?

Yes

Are sufficient details of methods and analysis provided to allow replication by others?

Partly

Reviewer Expertise:

Pending Claim, Coding ICD-10, ICD-9-CM, Design Web for early warning detection of pending claim, etc.

I confirm that I have read this submission and believe that I have an appropriate level of expertise to confirm that it is of an acceptable scientific standard, however I have significant reservations, as outlined above.

# Response

**Published:** 2026-08-26

**Authors:** Kori Puspita Ningsih

**Affiliations:** Public Health, Doctoral Program in Public Health, Faculty of Medicine, Universitas Sebelas Maret, Surakarta, 57126, Indonesia

Dear Reviewer,

 Thank you very much for your careful, comprehensive, and constructive review of our manuscript. We sincerely appreciate the time and expertise you have devoted to evaluating our work and for highlighting important areas that require further clarification and methodological strengthening.

 We have carefully noted all of your comments and recommendations. We are currently revising the manuscript accordingly and will address each point systematically, including clarification of the conceptual structure of the CCQAI–CM, the instrument-development process, expert-panel characteristics, the scope of validation, reliability analysis, scoring thresholds, sample characteristics, rater procedures, terminology, and the limitations and directions for further validation.

 Where the requested analyses can be appropriately conducted using the available data, we will incorporate them. Where they extend beyond the scope or available data of the present study, we will clarify the scope of the current research, moderate our claims where appropriate, and explicitly identify these issues as priorities for further validation.

 A detailed point-by-point response will accompany the revised manuscript.

 We sincerely thank you for these valuable recommendations, which will help us substantially strengthen the clarity, methodological transparency, and scientific positioning of the CCQAI–CM.

 With kind regards,

 The Authors
